# A Little Kills

**Published:** 1859-02

**Authors:** 


### A LITTLE KILLS.

Pope Adrian died by a gnat.
A Roman counsellor by a hair.
Anacreon, the Greek poet, by a grape-seed.
Charles the Sixth, by a mushroom.
Stephen Girrard, by a milk-cart.

Jacob Ridgeway, by a dray.

General Taylor, by a bowl of berries.

The Duke of Wellington, by a plate of venison.

Abbott Lawrence, by an injudicious change of clothing.

Rachel, the tragedienne, from want of an extra dress in the cars between New York and Boston.

Life, being hung on such little things, its preservation is a daily miracle; and that any of us should arrive at mature age is owing to the fact that there is an eye upon us which never sleeps, the eye of a Heavenly Father, whose loving kindness is over all his works—whose " mercies are new every morning, and fresh every evening."

